# Local Habitat Filtering Shapes Microbial Community Structure in Four Closely Spaced Lakes in the High Arctic

**DOI:** 10.3389/fmicb.2022.779505

**Published:** 2022-02-11

**Authors:** Catherine Marois, Catherine Girard, Yohanna Klanten, Warwick F. Vincent, Alexander I. Culley, Dermot Antoniades

**Affiliations:** ^1^Département de Biochimie, Microbiologie et Bio-Informatique, Université Laval, Québec, QC, Canada; ^2^Centre d’Études Nordiques (CEN), Université Laval, Québec, QC, Canada; ^3^Institut de Biologie Intégrative des Systèmes (IBIS), Université Laval, Québec, QC, Canada; ^4^Département des Sciences Fondamentales, Université du Québec à Chicoutimi, Chicoutimi, QC, Canada; ^5^Département de Géographie, Université Laval, Québec, QC, Canada; ^6^Département de Biologie, Université Laval, Québec, QC, Canada

**Keywords:** diversity, connectivity, predatory bacteria, Ellesmere Island, Stuckberry Valley, amplicon sequence variant (ASV), Arctic lake

## Abstract

Arctic lakes are experiencing increasingly shorter periods of ice cover due to accelerated warming at northern high latitudes. Given the control of ice cover thickness and duration over many limnological processes, these changes will have pervasive effects. However, due to their remote and extreme locations even first-order data on lake ecology is lacking for many ecosystems. The aim of this study was to characterize and compare the microbial communities of four closely spaced lakes in Stuckberry Valley (northern Ellesmere Island, Canadian Arctic Archipelago), in the coastal margin zone of the Last Ice Area, that differed in their physicochemical, morphological and catchment characteristics. We performed high-throughput amplicon sequencing of the V4 16S rRNA gene to provide inter- and intra-lake comparisons. Two deep (>25 m) and mostly oxygenated lakes showed highly similar community assemblages that were distinct from those of two shallower lakes (<10 m) with anoxic bottom waters. *Proteobacteria*, *Verrucomicrobia*, and *Planctomycetes* were the major phyla present in the four water bodies. One deep lake contained elevated proportions of *Cyanobacteria* and *Thaumarchaeota* that distinguished it from the others, while the shallow lakes had abundant communities of predatory bacteria, as well as microbes in their bottom waters that contribute to sulfur and methane cycles. Despite their proximity, our data suggest that local habitat filtering is the primary determinant of microbial diversity in these systems. This study provides the first detailed examination of the microbial assemblages of the Stuckberry lakes system, resulting in new insights into the microbial ecology of the High Arctic.

## Introduction

The Arctic is among the regions on Earth most affected by the rapid acceleration of global warming. It is estimated that air temperature is increasing more than twice as fast as the global average due to Arctic amplification (Meredith et al., 2019). The region is facing changes in its oceanic and atmospheric circulation, ice and snow extent and duration, permafrost, hydrology, vegetation, and carbon cycling (Wrona et al., 2016; Vincent, 2020; Cai et al., 2021). These changes have repercussions for ecosystem function at different spatial and temporal scales that are still not fully understood.

Lakes are sentinels of environmental change because they are integrators of watershed and airshed processes (e.g., plant and soil dynamics, nutrient loading, climate shifts, anthropogenic impacts; Williamson et al., 2008; Vincent, 2018). They are important and highly diverse features of the Arctic landscape, ranging from deep proglacial lakes to shallow thermokarst ponds (Pienitz et al., 2008). Due to warming climates, the abundance and area of glacial lakes are increasing (Shugar et al., 2020), while other types of lakes are disappearing (Smith et al., 2005). With their simplified food webs and unique physical and chemical characteristics (low water temperature, prolonged ice cover and highly seasonal photoperiods; Vincent, 2018), these extreme waterbodies represent key habitats that can be used to better understand the polar biome in transition.

Microorganisms dominate the food webs of Arctic lakes (Vincent, 2010), and their physiological diversity underpins the four major biogeochemical cycles that occur in aquatic ecosystems: carbon (including methane), sulfur, phosphorus, and nitrogen (Falkowski et al., 2008). Microbial distribution is determined by competition for heterogeneous niches created throughout the water column by chemical and physical gradients and other controlling forces such as predation and viral lysis (e.g., Shade et al., 2008; Comeau et al., 2012; Somers et al., 2020). Despite their extreme conditions, High Arctic Lakes host a relatively high diversity of microbial phyla, many of which are also found in temperate environments. For example, *Actinobacteria*, *Bacteroidetes*, *Cyanobacteria*, *Proteobacteria*, and *Verrucomicrobia* are phyla that are commonly found in temperate freshwater microbial communities (Zwart et al., 2002; Newton et al., 2011), but have also been identified in Arctic lakes such as Fuglebekken and Revvatnet (Norway) (Ntougias et al., 2016), Lake Hazen (Canada) (Cavaco et al., 2019), and Ward Hunt Lake (Canada) (Comte et al., 2018), as well as in Antarctic lakes (Byers Peninsula) (Picazo et al., 2019). Mat-producing and free-living (picoplanktonic) *Cyanobacteria* are the major bacterial primary producers in high-latitude lakes, and they conduct oxygenic photosynthesis (Vincent, 2018). Green and purple sulfur and non-sulfur bacteria carry out photosynthesis in low oxygen hypolimnia (Lizotte, 2008). Methanogens and methanotrophs, as well as sulfur reducers, sulfur oxidizers, and nitrifiers, are distributed above, across, or below oxyclines according to their specific redox requirements, for example as demonstrated by Schutte et al. (2016) in Potentilla Lake, a seasonally ice-covered dimictic lake in Greenland, and Vigneron et al. (2021) in meromictic Lake A in the Canadian High Arctic.

Microorganisms are responding rapidly to changing conditions in the warming Arctic (Yadav et al., 2019). For example, psychrophilic microorganisms may be replaced by psychrotrophic ones (Vincent, 2010), and the northward expansion of the boreal and tundra vegetation boundaries may contribute greater inputs of organic matter and increase lake productivity (Wrona et al., 2016). Climate change is also expected to modify microbially driven biogeochemical cycles. Indeed, mainly due to increased ice-free periods, perturbations have been observed, including the intensification of methane emissions (Tan and Zhuang, 2015) and the accumulation of reduced sulfur in sediments (Drevnick et al., 2010). In short, in addition to being key components of lake ecosystems, Arctic lacustrine microbial communities are greatly affected by climate change. However, the way they are affected, and the consequences of these changes, remain poorly understood. Although we were unable to determine the response of these microbial communities to climate change in this study, it is urgent to characterize the diversity of current communities before they are transformed.

To better understand microbial ecology in High Arctic lakes, we examined four lakes in Stuckberry Valley that are closely spaced and physicochemically and morphologically diverse (Top, Y, 2FB, and Bottom lakes). Located on the northern coast of Ellesmere Island (Nunavut, Canada), approximately 780 km from the North Pole, these lakes are under great pressure due to climate change (Mueller et al., 2009; White and Copland, 2018; Moore et al., 2019). They lie in the coastal margin zone of the Last Ice Area, which contains the thickest sea ice in the Arctic Ocean, but is now subject to rapid warming and ice loss. The vulnerability of this area to environmental change is evidenced by the recent loss of 43% of the Milne Ice Shelf, the last intact Canadian ice shelf (Vincent and Mueller, 2020), and the over 90% reduction of the coastal ice shelves of Ellesmere Island since the beginning of the twentieth century (Copland and Mueller, 2017). The four Stuckberry Valley lakes were successively isolated from the Arctic Ocean by glacioisostatic rebound, a process in which glacial retreat leads to rising of the Earth’s crust, trapping water, and thus creating lakes.

Previous comparisons of morphologically distinct lakes in the Byers Peninsula (Antarctica) have shown that nutrients, pigments, and bacterial communities follow a gradient from inland to coastal sites (Rochera et al., 2013) and that this shift was reflected in predicted microbial metabolism (Picazo et al., 2021). The objective of this study was to characterize and compare the microbial communities within four lacustrine ecosystems in the Canadian High Arctic. We hypothesized that local habitat filtering based on the distinct chemical and physical gradients, as well as the unique catchment and morphological characteristics of each waterbody, would be the primary determinant of microbial diversity despite their close proximity. To test this hypothesis, we performed deep sequencing of the V4 region of the 16S rRNA gene on samples collected at several depths in each water column. We then determined richness and evenness within communities, the identified dominant phyla, and similarities in microbial community composition in the context of physicochemical water column variability. These data also enabled us to measure the impacts of local and regional forcing, and thus lay the foundation for a better understanding of the ecological impacts of a warming Arctic.

## Results and Discussion

### Limnological Characteristics

**FIGURE 1:**
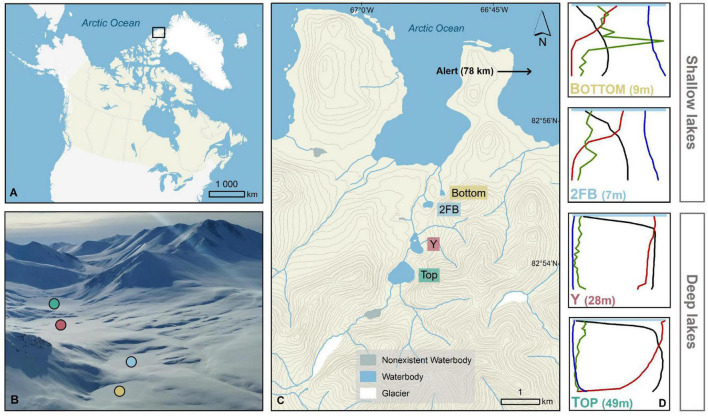
Stuckberry Valley lakes. **(A)** Stuckberry Valley is located on the northern coast of Ellesmere Island on the edge of the Arctic Ocean **(B)** Aerial photograph of the four lakes surrounded by mountains. In late May, lakes were still covered with ice and snow (photo credit: Addison Gilpin-Payne). **(C)** The valley includes four lakes. **(D)** Chemical and physical gradients in the water columns of the four Stuckberry Valley lakes. For all four lakes, the maximum depth is in parentheses. Each profile parameter is associated with a color: green, chlorophyll *a*; blue, specific conductivity; black, temperature; red, dissolved oxygen. Complete profiles with values are available in Supplementary Figure 1. Maps were created using ESRI ArcMap (v.10.7). The digital elevation model comes from Natural Resources Canada (2001) and the boundary files are from Statistics Canada (2016).

The Stuckberry Valley lakes fell into two categories based on their limnological and physical characteristics: deep and shallow (Figure 1). The deep lakes (Top and Y) had maximum depths of 49 and 28 m, respectively, and larger watershed areas (14.4 and 17.5 km^2^). At the time of sampling, dissolved oxygen (DO,% saturation) levels were between 85 and 100% at the surface and declined steadily with depth, reaching hypoxic conditions in Top Lake (5.6%) at 45 m (Supplementary Figure 1). Hypoxic conditions were not reached in Y Lake. Both lakes had low specific conductivities (mean water column values of 143 and 139 μScm^–1^ for Top and Y lakes, respectively) and DOC concentrations (respective means of 0.4 and 0.5 mgL^–1^). A stream directly connected Top Lake with Y Lake, and is likely a major reason for the similar characteristics of the two lakes. The shallow lakes (2FB and Bottom) were less than 10 m deep and had smaller watershed areas (each ∼0.4 km^2^). They had steep oxyclines, with DO concentrations of ∼50% just below the surface that decreased sharply at a depth of ∼2 m before becoming anoxic near the lake bottoms. The shallow lakes had notably higher specific conductivities (mean water column values of 387 and 401 μScm^–1^ in 2FB and Bottom lakes, respectively) and DOC concentrations (means = 1.3 and 2.2 mgL^–1^, respectively) than the deeper lakes. The deeper, more dilute lakes were also more transparent, with 1% of solar radiation that arrived at the surface of the water (i.e., below the ice and snow) reaching 30.8 and 19.3 m in Top and Y lakes, but only 5.9 and 6.4 m in 2FB and Bottom lakes, respectively. Over 0.1% of PAR reached the sediment surface in all lakes except Top Lake.

Although the lakes were separated into two groups (shallow and deep) based on morphological properties, one of the shallow lakes (Bottom Lake) had unique characteristics, including a chlorophyll *a* peak at 4 m and higher total phosphorus (TP) and nitrogen (TN) concentrations (Table 1). In contrast, nutrient concentrations in Top, Y, and 2FB were typical of Arctic oligotrophic lakes (Lizotte, 2008). Viral particle and heterotrophic bacterial counts from flow cytometry were approximately five and four times higher, respectively, in Bottom Lake than in the others. We also observed a peak in photosynthetic eukaryotes at 4 m in Bottom Lake (Table 1), which was much deeper in the water column relative to the other lakes.

**TABLE 1:** Limnological characteristics of sampled depths for each studied lake.

		Limnological properties						Microbial abundance				
Lake	Depth	DOC	TP	TN	Chlorophyll *a*	SO_4_^2–^	PAR	Photosynthetic eukaryotes	Photosynthetic bacteria	Heterotrophic bacteria	Viruses	VPR
	(m)	(mgL^–1^)	(μgL^–1^)	(μgL^−1^)	(μgL^–1^)	(mgL^–1^)	%	(10^3^mL^–1^)	(10^3^mL^–1^)	(10^3^mL^–1^)	(10^3^mL^–1^)	
Top	0	0.3	12.5	236	1.08	22.5	100	6.1 (7)	15.3 (16)	338.9 (15)	1137.9 (65)	3.2
	10	0.3	7.8	218	1.02	21.9	NA	4.1 (16)	6.8 (3)	172.3 (15)	1125.6 (19)	6.3
	20	0.3	3.2	231	0.60	22.1	NA	1.6 (18)	4.9 (10)	241.1 (5)	879.6 (17)	3.6
	45	0.7	5.6	285	0.42	22.1	NA	1.2 (3)	2.3 (1)	333.9 (8)	807.3 (7)	2.4
Y	0	0.9	4.0	237	0.84	23.0	100	2.4 (0)	1.0 (3)	359.5 (1)	944.0 (21)	2.6
	10	0.3	7.1	236	0.53	21.6	NA	1.2 (15)	1.5 (26)	349.2 (1)	1720.3 (47)	4.9
	25	0.6	4.4	280	1.27	21.9	NA	1.2 (12)	9.2 (8)	275.3 (19)	1147.8 (59)	4.0
2FB	0	1.4	8.7	154	1.91	42.8	100	1.8 (20)	0.6 (22)	358.5 (22)	2153.3 (10)	6,0
	2	1.4	8.5	176	1.74	42.6	22.31	2.9 (38)	0.4 (28)	327.6 (8)	2791.4 (46)	8.5
	3	1.2	4.4	183	1.39	41.2	14.23	2.4 (61)	0.7 (7)	314.2 (39)	2384.3 (29)	7.6
	5	1.3	7.1	235	0.82	38.6	3.46	2.7 (13)	11.7 (35)	419.1 (15)	1570.5 (47)	3.6
Bottom	0	2.1	23.8	310	2.54	32.9	100	6.8 (23)	3.4 (4)	1027.7 (2)	8084.5 (13)	7.8
	3	2.1	19.3	329	4.10	33.0	6.93	7.4 (29)	2.3 (16)	1056.0 (19)	7640.5 (9)	7.2
	4	1.9	22.8	362	8.78	30.9	3.80	10.4 (0)	11.7 (11)	1581.2 (10)	7215.8 (3)	4.5
	7.5	2.4	37.5	1020	1.13	20.1	0.15	1.8 (43)	103.6 (14)	936.4 (4)	7215.1 (6)	6.9

*The limits of detection were 0.1 mgL^–1^ for dissolved organic matter (DOC), 0.5 μgL^–1^ for total phosphorus (TP), 15 μgL^–1^ for total nitrogen (TN), 0.01 μgL^–1^ for chlorophyll a and 0.1 mgL^–1^ for the sulfate concentration (SO_4_^2–^). Photosynthetically active radiation (PAR) could not be measured (NA) below a depth of 7.5 m. The virus-to-prokaryote ratio (VPR) was calculated by dividing the virus counts by the sum of photosynthetic and heterotrophic bacteria counts. Values represent the mean of two replicates with percent coefficient of variation (CV) in parentheses.*

In all lakes, viral particles were approximately two to nine times more abundant than cellular microbes (Table 1), which corresponds with the averages observed in Arctic and Antarctic lakes (Sawstrom et al., 2008; Rochera et al., 2017). The virus-to-prokaryote ratio (VPR) can be influenced by multiple biological factors that affect viral and prokaryotic abundances (e.g., depth, season) (Wommack and Colwell, 2000), as well as methodological biases such as a high abundance of viruses (virus-like particles) that infect eukaryotes.

### Intra-Lake Comparisons

All four lakes had chemical and physical gradients in their water column (Figure 1 and Table 1). This heterogeneity suggests that there were distinct ecological niches for organisms (e.g., Shade et al., 2008; Comeau et al., 2012; Somers et al., 2020). Others have observed distinct bacterial communities at different depths in both non-Arctic and Arctic lakes with gradients (e.g., Shade et al., 2008; Schutte et al., 2016; Vigneron et al., 2021) and our results are consistent with these studies.

We investigated whether the microbial community composition, which contained a total of 4,155 unique amplicon sequence variants (ASVs) (Supplementary Table 1), reflected the observed partitioning along depth and chemical gradients. We did this by estimating the microbial diversity and characterizing the major phyla at each depth for the four lakes. We also identified features (or microbial biomarkers), which are differentially abundant features that most likely explain the differences noted between lakes, using linear discriminant analysis effect size (LEfSe) analysis (Segata et al., 2011; see below). A total of 20 features were identified among the four lakes (5, 3, 8, and 4 in Top, Y, 2FB and Bottom lakes, respectively).

**FIGURE 2:**
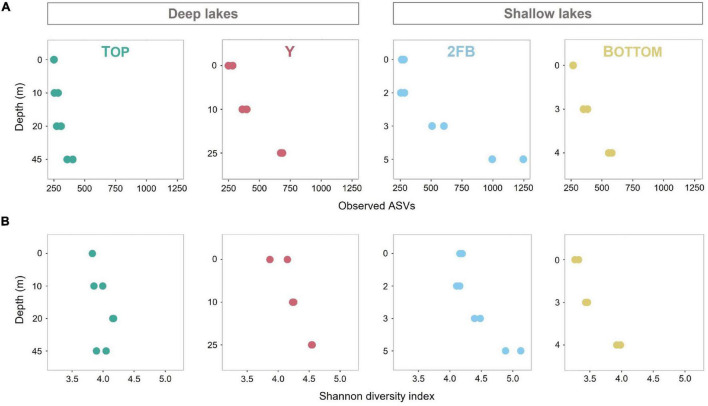
Observed diversity and Shannon diversity in all four Stuckberry Valley lakes. **(A)** Microbial richness calculated with Observed species index. **(B)** Microbial richness and evenness calculated with Shannon diversity index. Colors are associated with each lake: turquoise, Top; pink, Y; blue, 2FB; yellow, Bottom. Note that each sample had two replicates (except 0 m in Top Lake) and the changing graduations of the y-axis established according to the maximum depth of the four lakes.

ASV richness increased significantly with depth in Y, 2FB and Bottom lakes (Observed species index, Figure 2A). 2FB Lake had the richest microbial communities with 1,706 different ASVs, vs. 610, 1,093, and 909 for Top, Y and Bottom lakes, respectively. When evenness was considered (Shannon diversity index), only microbial communities in the bottom waters of the shallow lakes were significantly more diverse compared to the surface (Figure 2B). In Top Lake, we observed a decrease in evenness at depths of 20 m and below, while richness increased. Overall, the microbial community evenness was lower in Bottom than in 2FB and Y lakes, while richness remained similar; however, none of these differences were significant. One explanation for increases in microbial diversity with depth may be that the ice cover prevents wind-driven mixing, thereby allowing chemical gradients to persist in the deeper waters, favoring a wider range of species and fewer dominants. A similar trend was observed by Schutte et al. (2016), who reported richer and more even communities at depth in Potentilla Lake in Greenland, which had strongly stratified oxygen conditions similar to 2FB and Bottom lakes. Driven by the internal waves in ice-covered lakes, the resuspension of sediments, including microbes, could also explain the increase in microbial diversity with depth (Bouffard et al., 2016).

**FIGURE 3:**
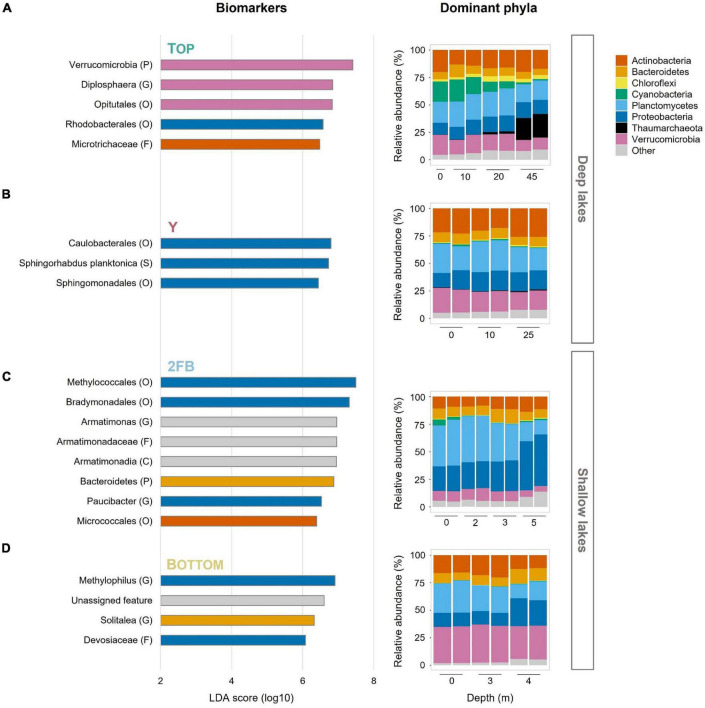
Features and relative abundances of major phyla in the four Stuckberry Valley lakes. **(A)** Top Lake, **(B)** Y Lake, **(C)** 2FB Lake, and **(D)** Bottom Lake. The linear discriminant analysis (LDA) score (log 10) was used to estimate the size effect of each biomarker. The letters in parentheses correspond to the taxonomic rank (P, phyla; C, class; O, order; F, family; G, genus; S, species) and colors correspond to phyla. The color of the lake name is consistent with the map in Figure 1. In Bottom Lake, the unassigned feature represents an ASV without any taxonomic assignment. In the relative abundance graphs, phyla that represent less than 5% of reads in at least one sample were grouped together as “Other.” Note that each sample had two replicates (except 0 m in Top Lake).

Hallmark phyla of freshwater environments were all observed in the Stuckberry Lakes system, including *Actinobacteria*, *Bacteroidetes*, *Cyanobacteria*, *Proteobacteria*, and *Verrucomicrobia* (Figure 3). *Actinobacteria* and *Bacteroidetes* maintained relatively constant relative abundances throughout the water columns of all the lakes, averaging 16 and 9% of reads, respectively. These phyla are widespread in freshwater lakes (Zwart et al., 2002; Newton et al., 2011), including High Arctic (Ntougias et al., 2016; Comte et al., 2018; Cavaco et al., 2019) and Antarctic lakes (Picazo et al., 2019).

#### Top Lake

*Cyanobacteria*, often major primary producers in Arctic and Antarctic inland waters (Vincent, 2018), were relatively more abundant at the surface of Top Lake than in the other waterbodies (Figure 3A). Picocyanobacteria are often the dominant phytoplankton in terms of cell concentrations in cold, oligotrophic lakes, where their abundance can be increased by nutrient supply, (e.g., by mixing and nutrient entrainment in High Arctic Lake A; Veillette et al., 2011). The relatively higher TP yet overall oligotrophic conditions (Table 1) combined with high water transparency (Klanten et al., 2021) may have favored their relative abundance in Top Lake. *Chloroflexi* were mostly found at 20 m in Top Lake. This phylum is known to include aerobic phototrophs that do not produce oxygen but use bacteriochlorophyll *a* to perform anoxygenic photosynthesis and photoheterotrophy (Ward et al., 2018). Representatives are commonly found in northern lakes with high light penetration and oligotrophic conditions (Fauteux et al., 2015), and in the microbial mats that often coat the sediments of high latitude waterbodies (Vigneron et al., 2018). Similarly, *Rhodobacterales* were identified as important features in Top Lake (Figure 3A). This order also contains aerobic anoxygenic phototrophs (Imhoff et al., 2018) and chemoheterotrophs (Kersters et al., 2006). Both *Rhodobacterales* and *Chloroflexi* may contribute to total primary production and carbon fluxes in this deep oligotrophic lake.

We observed a high relative abundance of *Thaumarchaeota* in the hypolimnion of Top Lake. The fact that this is the only archaeal phylum identified in our dataset is likely because our marker gene approach targeted a bacteria-specific gene (V4 region of 16S). These ammonia-oxidizing archaea prefer aquatic habitats with low dissolved oxygen, pH, nutrients, and light (Erguder et al., 2009; Hatzenpichler, 2012; Auguet and Casamayor, 2013; Hollibaugh, 2017; Juottonen et al., 2020). These conditions occurred only at the bottom of Top Lake (Figure 1 and Table 1), in which pH was 7.59 at 45 m (in 2019) and photosynthetically active radiation (PAR) was ∼0% at 45 m (Klanten et al., 2021). The greater pH (7.94 and 8.15 for 2FB and Bottom lakes, respectively) and lower PAR values under the ice (3.46% at 5 m and 0.15% at 7 m for 2FB and Bottom lakes, respectively) may explain why *Thaumarchaeota* relative abundance was not as high at the bottom of 2FB and Bottom lakes. The importance of this group as ammonia oxidizers has been shown in multiple studies (Erguder et al., 2009; Hatzenpichler, 2012; Stahl and de la Torre, 2012), and their ammonium oxidation potential has been previously recorded across the oxycline of High Arctic meromictic lakes (Pouliot et al., 2009). The results from Top Lake suggest that *Thaumarchaeota* drove nitrogen cycling in this part of the lake. *Diplosphaera*, an abundant feature in Top Lake (Figure 3A), has been found to be capable of nitrogen fixation in microaerophilic conditions (Wertz et al., 2012). While our taxonomic approach cannot confirm this, the overrepresentation of this taxa suggests it may contribute to nitrogen cycling in the lake.

*Verrucomicrobia* and its representatives (e.g., *Opitutales*) were identified as features only in Top Lake (Figure 3A). *Verrucomicrobia* is a diverse phylum that includes members that play a role in nutrient and carbon cycling in eutrophic and DOC-rich lakes (He et al., 2017). Their relative abundance here in perennially cold, oligotrophic, DOC-poor Top Lake extends the range of known habitats for these bacteria.

#### Y Lake

In Y Lake, all the major phyla had relatively constant relative abundances throughout the water column, reflecting the minimal variations in the limnological profile (Figure 3B). Here, three features from the *Alphaproteobacteria* were identified: *Caulobacterales, Sphingorhabdus planktonica*, and Sphingomonadales).

#### 2FB Lake

*Planctomycetes* were present in all four Stuckberry Lakes (Figure 3), as expected given the wide freshwater distribution of this group (Zwart et al., 2002; Newton et al., 2011). These heterotrophs have various important ecosystem functions, including contributing to the degradation of complex organic compounds (Schlesner et al., 2004; Fuchsman et al., 2012), and they are mostly found in the hypolimnia of deep oxygenated lakes (Okazaki and Nakano, 2016; Karlov et al., 2017; Okazaki et al., 2017; Storesund et al., 2020). By contrast, 2FB Lake, where *Planctomycetes* reached their greatest relative abundances in our dataset (40%) (Figure 3C), was shallow and hypoxic. However, there appeared to be a relationship between *Planctomycetes* communities and DO concentrations in Stuckberry Valley lakes, as proportions were relatively constant throughout the water columns of the deep, oxygenated lakes, and declined markedly below the oxyclines of the two shallow lakes.

*Proteobacteria* constitute the largest and most phenotypically diverse phylum of bacteria. Members of this taxon are essential contributors to key lacustrine biogeochemical cycles (Kersters et al., 2006). This explains their dominance among the features that are responsible for differences between lakes (Figure 3). Some of these bacteria had high relative abundances in the Stuckberry Valley lakes, but mainly in the deep anoxic waters of 2FB Lake (46%) (Figure 3C). Key *Proteobacteria* in this shallow lake are discussed below.

In 2FB Lake, as in Bottom Lake, sulfate concentrations decreased with depth (Table 1) suggesting the presence of sulfate-reducing bacteria. This is consistent with the pronounced odor of hydrogen sulfide detected in the anoxic deep waters during sampling. *Deltaproteobacteria*, which include sulfate-reducing bacteria (e.g., *Desulfuromonadales)* (Kersters et al., 2006), were present in the hypolimnia of 2FB and Bottom lakes (Supplementary Figure 2A). These bacteria have also been shown to dominate the lower water columns in polar lakes with anoxic bottoms, such as Arctic Potentilla Lake (Schutte et al., 2016), and Antarctic Lake Fryxell (Karr et al., 2005).

**FIGURE 4:**
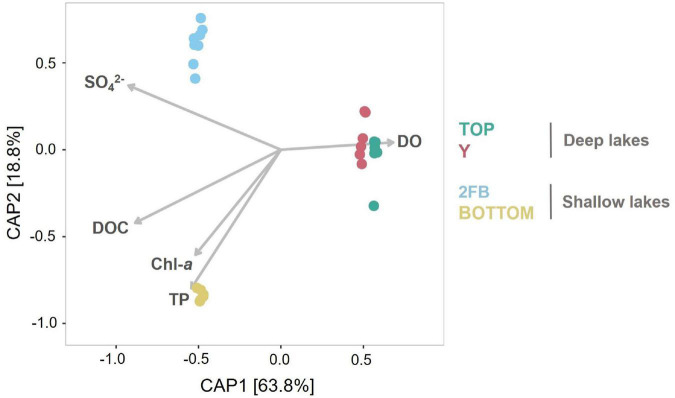
Constrained analysis of principal coordinates of the lake microbial community composition constrained to five environmental variables. Vectors for dissolved oxygen (DO), dissolved organic carbon (DOC), total phosphorus (TP), chlorophyll a (Chl-*a*) and the sulfate concentrations (SO_4_^2–^) illustrate correlations between samples and each of these limnological properties. Colors are associated with each lake: turquoise, Top; pink, Y; blue, 2FB; yellow, Bottom. Note that each sample had two replicates (except 0 m in Top Lake).

*Gammaproteobacteria* (35%) were among the *Proteobacteria* that dominated the bottom of 2FB Lake. These included *Methylococcales*, which use methane and methanol as carbon and energy sources (Kersters et al., 2006; Figure 3C and Supplementary Figure 2B), and *Chromatiales*, which are photosynthetic purple sulfur bacteria that typically grow under anoxic conditions (Kersters et al., 2006). The high relative abundance of sulfate-reducing (Supplementary Figure 2A) and purple sulfur bacteria (*Chromatiales*) (Supplementary Figure 2B) suggest that these groups were important contributors to the cycling of sulfur in the anoxic bottom waters of 2FB Lake. The major role of the sulfur cycle in this lake is also indicated by the association of its microbial communities with the sulfate vector in the ordination analysis (Figure 4). Schutte et al. (2016) found a similar distribution in Potentilla Lake, where microbes involved in the sulfur and methane cycles were most abundant below the oxycline, where they likely compete for resources. Despite the presence of methane oxidizers, we did not detect methanogenic archaea. This may reflect limitations of the primer set used, or competitive exclusion of methanogens by sulfate reducers, as observed elsewhere (e.g., Pester et al., 2012). It is also possible that methanogenesis is restricted to the bottom sediments. In High Arctic Ward Hunt Lake, for example, methanogenic Archaea were detected in microbial mats that coated the bottom sediments and experienced anoxic conditions in winter (Mohit et al., 2017).

Viruses and eukaryotic grazers are well-known agents of bacterial mortality in aquatic ecosystems (Fuhrman and Noble, 1995; Ram et al., 2014). However, additional sources of top-down control, such as predatory bacteria, have remained relatively poorly characterized in freshwater lakes. In Stuckberry Valley, we observed high relative abundances of the *Deltaproteobacteria* orders *Bdellovibrionales*, and *Bradymonadales* in 2FB Lake (Supplementary Figure 2A), which are known predators of other Gram-negative bacteria (Mu et al., 2020). Interestingly, higher relative abundances of these taxa appeared to correspond with lower counts of heterotrophic bacteria which include their prey (Kersters et al., 2006), and vice versa (Table 1). The identification of *Bradymonadales* as a features also supports the biological significance of these taxa as a top-down control in this shallow lake (Figure 3C). The opportunistic predators *Myxococcales* (Perez et al., 2016) were also detected in the bottom waters of the shallow lakes (Supplementary Figure 2A), and their relative abundances proportionally increased as *Bdellovibrionales* and *Bradymonadales* decreased. Abundant communities of predatory bacteria in lakes have been observed elsewhere, including in Lake Geneva (Paix et al., 2019; Ezzedine et al., 2020). The data from Lake Geneva and from our study suggest that the community composition of prey bacteria (Chen et al., 2011) and the competition between predators (Chase et al., 2002; Johnke et al., 2017) may shape the community structure of these predators and their prey. High Arctic lakes are also known to contain a wide range of phagotrophic protists as well as rotifers (e.g., ten species of rotifers and numerous mixotrophic and heterotrophic protists in ice-covered Ward Hunt Lake; Bégin et al., 2021a,b), which likely exert a top-down grazing control on the bacterial communities. However, eukaryotic grazers are likely to have a minimal grazing impact in anoxic waters (e.g., Oikonomou et al., 2014), where predatory bacteria and viruses could play the greater role.

#### Bottom Lake

*Verrucomicrobia* were present in all Stuckberry Valley lakes at all depths, despite the pronounced limnological differences. These bacteria are common in freshwater ecosystems (Zwart et al., 2002; Newton et al., 2011), including in the Arctic (Ntougias et al., 2016; Comte et al., 2018; Cavaco et al., 2019), and are found throughout water columns regardless of nutrient concentrations (Kolmonen et al., 2011). This group includes aerobic, facultative anaerobic, and obligate anaerobic heterotrophs. With their high potential for degrading polysaccharides, they can use multiple carbon sources (He et al., 2017). This metabolic plasticity likely explains why they were so widely distributed in Stuckberry Valley lakes and why they were identified as features in Top Lake (Figure 3)—this is likely due to *Diplosphaera* and its order *Opitutales*, which have also been identified as features of this lake. However, we observed the highest relative abundances of *Verrucomicrobia* in Bottom Lake (33%) (Figure 3D), where phosphorus and nitrogen concentrations reached their maxima in our dataset (Table 1) and where the microbial assemblages were associated with the TP vector (Figure 4). This is consistent with the prevalence of this phylum in high-latitude lakes such as Lake Siggeforasjön (Sweden), Lake Vesijärvi (Finland), and Lake Joutikas (Finland) (Kolmonen et al., 2004; Lindstrom et al., 2004; Haukka et al., 2006). With longer ice-free periods due to warming (Magnuson et al., 2000; Vincent, 2018) that will increase organic carbon and nutrient inputs into lakes (Luoto et al., 2019), we hypothesize that generalist taxa such as *Verrucomicrobia* will make up a larger portion of the bacterial flora in Arctic aquatic ecosystems.

As in 2FB Lake, the high abundance of sulfate-reducing bacteria and decrease in the sulfate concentration in Bottom Lake (Table 1 and Supplementary Figure 2A) suggests that sulfur was being actively metabolized at depth. The differentially abundant taxa *Methylophilus* and *Devosiaceae* (Figure 3D) may contribute to the carbon (methane) and nitrogen cycles, respectively (Kersters et al., 2006). Although microbial activities can often be inferred from taxonomy, we did not measure transcription or microbial processes directly and note that our inferences are limited to the genetic potential for biogeochemical functions.

### Inter-Lake Comparisons

Constrained Analysis of Principal Coordinates (CAP) showed that differences between lakes explained a high proportion of community composition variance (*R*^2^ = 0.90), with non-significant within-lake dispersal (Figure 4). A high proportion of total variance (63.9%) was explained by the first axis, and samples were closely grouped on this axis according to lake type (deep vs. shallow). However, while samples from Top and Y lakes overlapped on the second axis (18.8% of variance explained), microbial communities in Bottom and 2FB lakes showed clear separation. This separation appears driven by dissolved oxygen (Supplementary Figure 3). In Stuckberry Valley, the outflow stream associated with Top Lake flows into Y Lake, which likely contributes to their similar microbial communities. Studies of microbial connectivity across subarctic and Arctic watersheds have shown that some microbial communities in upstream habitats, such as snow and rivers, were also present in downstream microbiomes, suggesting a landscape-level microbial seeding effect (Ruiz-González et al., 2015; Cavaco et al., 2019). In some locations, up to 30% of phylotypes were shared along the hydrologic continuum (Comte et al., 2018). In contrast to Top and Y lakes, 2FB and Bottom lakes have no hydrological connection (Figure 1), and this likely contributes to their marked separation. Lake metabolic processes are depth-dependent, with smaller ecosystems showing greater variability than larger ecosystems (Staehr et al., 2012), further explaining why the shallow lakes sampled in the present study had fewer similarities between them. In small productive lakes, these depth-dependent changes can be accompanied by sharp vertical gradients in chemical conditions (chemoclines), which increase the diversity of potential bacterial niches, and thus variability, within and between lakes.

Although the Stuckberry Valley lakes could be easily divided into two groups according to their limnological and physical properties (as suggested in Supplementary Figure 3), no such clear divisions were evident in their microbial community compositions due to differing relative abundances of major phyla and microbial features (Figure 3). Previous studies of lakes with distinct limnological characteristics in larger regions (Lindstrom, 2000; Van der Gucht et al., 2001, 2005; Somers et al., 2020) have reached similar conclusions about the primary role of physicochemical and morphological parameters in determining microbial communities. Those studies identified patterns like those observed in Figure 4, where samples clustered by lake type (deep vs. shallow). However, within these broader trends, our study found that each Stuckberry Valley lake also had an individual microbial community structure that was influenced by contrasting environmental conditions within the lakes. This is similar to observations in a study of 67 Finnish lakes, which suggests that individual responses toward environmental factors may occur among the bacterioplankton (Kolmonen et al., 2011).

## Conclusion

The northern coast of Ellesmere Island is undergoing fundamental changes due to accelerated regional warming. The lakes of Stuckberry Valley in this region are natural laboratories to better understand the impacts of climate change on aquatic ecosystems in the High Arctic. The limnological and microbial stratification we observed in all lakes is likely linked to ice cover which prevents mixing of the water column, and as ice cover decreases in thickness and duration with climate change, vertical stratification is likely to weaken, altering microbial communities at each depth. For the ensemble of Stuckberry Valley lakes, a key driver of microbial community structure appears to be local habitat filtering, i.e., the selection of taxa by unique characteristics of each catchment, and the physical and chemical environment of each lake. Examples include the high relative abundance of *Cyanobacteria* and *Thaumarchaeota* at the surface and at depth, respectively, in Top Lake, the high relative abundance of predatory bacteria in 2FB Lake, and the dominance of *Verrucomicrobia* throughout the Bottom Lake water column. Although more direct measurements of microbial function and more detailed molecular studies are needed to fully elucidate the microbial ecology of this system, these data establish an important baseline characterization for lakes in this remote, extreme, and vulnerable area.

## Materials and Methods

### Study Site

Stuckberry Valley (82° 54′N, 66° 56′W) is located on the northern coast of Ellesmere Island in the Canadian High Arctic (Figure 1). The four study sites (unofficially named Top, Y, 2FB, and Bottom lakes) were formed gradually by glacioisostatic rebound following glacial retreat (Bednarski, 1986; Dyke, 2004). Top Lake is the highest above sea level (56 m asl) and Bottom Lake is the closest to the ocean (31 m asl). About 4 km separates the far end of Top Lake from the opposite end of Bottom Lake. Top Lake, whose catchment contains a small glacier remnant, has an outlet stream that flows into Y Lake, while 2FB and Bottom are isolated from the other lakes and from each other. Y, 2FB, and Bottom lakes have outlets that flow directly into the Arctic Ocean. Based on regional emergence curves, the four lakes were formed approximately 5.6, 5.4, 4.0, and 4.0 ka calibrated years before present (cal BP), respectively (Klanten et al., 2021). The two oldest lakes (Top and Y) are deeper (49 and 28 m, respectively) and mostly oxygenated throughout their water columns. The two younger lakes (Bottom and 2FB) are shallow (< 10 m) and have anoxic bottom waters (Klanten et al., 2021). The deeper lakes are located at the head of the Stuckberry Valley, and presumably have different hydrological and nutrient inputs than the shallower lakes located at the mouth of the valley, closer to the ocean (Figure 1).

The nearest weather station is located at Alert (78 km to the east, Ellesmere Island). From 1981 to 2010, the annual average air temperature was –17.7°C (mean minimum in February of –37°C and mean maximum in July of 6.1°C), and annual precipitation averaged 158 mm. From November to May, the average snow cover reached 33 cm (Environment Canada).^1^ The polar night of continuous total darkness occurs from October to late February, while the polar summer of continuous daylight lasts from April to August.

### Sampling

Samples were collected from May 28th to June 3rd, 2018. Parameters for physicochemical profiles of the water column were measured using an EXO2 Multiparameter Sonde (YSI). Temperature, specific conductivity, chlorophyll *a* fluorescence, and dissolved oxygen profiles are summarily presented in Figure 1 (complete profiles available in Supplementary Figure 1). Photosynthetically active radiation (PAR) was measured using a cosine-corrected underwater quantum sensor (Li-189, LI-CO), as described in Klanten et al. (2021). The full data set for physical and chemical variables is archived in Antoniades et al. (2021).

Three 20-cm-diameter holes (1 m apart) were bored through the snow and ice cover with a manual ice auger at the deepest known point of each lake. The ice on Top Lake was 111 cm thick, while it was 90 cm for the other three lakes. Snow was more variable, with snow covers of 51, 73, 60, and 69 cm depth on Top, Y, 2FB and Bottom lakes, respectively. Water samples were collected in triplicate with a 6.2L-Kemmerer bottle at multiple depths within the water column that were selected according to the physicochemical profiles (Figure 1 and Supplementary Figure 1). Samples were stored in cubitainers that had been previously washed with 2% (vol/vol) Contrad™ 70 liquid detergent (DeconLabs), 10% (vol/vol) ACS-grade HCl (Sigma-Aldrich), and distilled water, and then rinsed three times with water from the lake. Three to four depths were sampled in each lake (0, 10, 20, and 45 m for Top Lake; 0, 10, and 25 m for Y Lake; 0, 2, 3, and 5 m for 2FB Lake; 0, 3, 4, and 7.5 m for Bottom Lake). Cubitainers were kept in the dark during sampling and transportation to the field laboratory.

Within 20 h of sampling, whole water samples were filtered at the field camp on Sterivex™ 0.22 μm capsule filters (Millipore) using a Masterflex^®^ Peristaltic Tubing Pump (Cole-Parmer) and Contrad-washed tubing. Samples were preserved by adding 2 mL RNA*later*™ Solution (Thermo Fisher Scientific) to the filters and then frozen. They were stored subsequently at –80°C once back from the field until extraction.

Water subsamples were mixed with EM grade glutaraldehyde (Canemco) to obtain a final concentration of 1% (vol/vol) for flow cytometry counts, which were analyzed as described in Belzile et al. (2008) and Brussaard et al. (2010). For dissolved organic carbon (DOC), dissolved inorganic carbon (DIC), particulate organic carbon (POC), particulate organic nitrogen (PON), total phosphorus (TP), total nitrogen (TN), major ions and metals analyses, subsamples of water were collected, treated, and stored as described in Klanten et al. (2021). Partial data are listed in Table 1, with complete data available in Klanten et al. (2021).

### Nucleic Acid Extraction and Processing

After removing RNA*later*™ Solution from Sterivex™ filters, nucleic acids were extracted using the AllPrep DNA/RNA Mini kit (QIAGEN). The manufacturer’s protocol was modified as described in Cruaud et al. (2017). DNA concentrations were quantified using the Qubit 3.0 Fluorometer (Thermo Fisher Scientific) as per the manufacturer’s protocol. Out of three replicates, the two with the highest DNA concentrations were used for the next steps. Library preparation and sequencing were performed by the Plateforme d’analyse génomique at the Institut de biologie intégrative et des systèmes (IBIS, Université Laval, Québec, Canada). The V4 region of the 16S rRNA gene was amplified by a 2-step PCR using the 515F forward primer (5′-GTGYCAGCMGCCGCGGTAA-3′) (Parada et al., 2016), the 806R reverse primer (5′-GGACTACNVGGGTWTCTAAT-3′) (Apprill et al., 2015), and the Q5 High-fidelity polymerase (NEB). Amplicons were purified on sparQ PureMag beads (QuantaBio) and sequenced on an Illumina MiSeq by paired-end sequencing (2 × 300 pb). The total sequencing yield was 8,447,001 reads (Supplementary Table 1).

### Sequencing Processing

Sequences were processed using the DADA2 package (v.1.14.0) (Callahan et al., 2016) in R (v.3.5.0) (R Core Team, 2020). Within the package, reads were subjected to quality filtering, trimming, error-rate learning, dereplication, amplicon sequence variant (ASV) inference (Callahan et al., 2017), paired-read merging, chimera removal and taxonomy assignment. The default parameters were used except for the *filterAndTrim()* function with a *truncLen* = (220,195) and *trimLeft* = (19,20). Taxonomy was assigned using the SILVA reference database (v.132) (Quast et al., 2013; Yilmaz et al., 2014). 30 samples were sequenced in total, but due to low counts of initial reads (< 2,000 reads compared to > 240,000 reads for others) (Supplementary Table 1), three were discarded (Top_0B, Bot_ 7.5A and Bot_7.5B). The DNA extracts from the two replicates at 7.5 m from Bottom Lake could not be re-sequenced and compared to the other samples due to the different methods that should have been used to remove PCR inhibitors.

Species richness (α diversity) estimates were calculated and plotted using the *plot_richness()* function with the Observed and Shannon diversity metrics in the package phyloseq in R (v.1.32.0) (McMurdie and Holmes, 2013). The Observed metric considers the numbers of different ASVs (richness) and the Shannon diversity index estimates the ASV richness and evenness (Hill et al., 2003). For both metrics, a *t*-test was used to compare surface and bottom water diversity estimates using the *T.TEST* formula (two-sample assuming equal variances) in Microsoft^®^ Excel^®^ (Microsoft 365; v. 2103) for all lakes separately except for Top Lake. The test could not be performed for Top Lake because there was only a single surface sample. Grouped samples within each lake were compared to others using a Kruskal-Wallis test [stats{}; v.4.0.2] (R Core Team, 2020) followed by a Dunn’s test using the FSA package (v.0.8.30) (Ogle et al., 2020), since parametric tests could not be carried out. The assumptions of normality and homoscedasticity were tested with Shapiro-Wilk and Bartlett’s tests (stats{}). The data met the assumption of normality but not homoscedasticity.

A bar plot was produced using ggplot2{} (v.3.2.2) (Wickham, 2016). The ASV table was converted to relative abundances and manipulated using tidyr{} (v.1.1.2) (Wickham, 2020), dplyr{} (v.1.0.2) (Wickham et al., 2020) and tibble{} (v.3.0.3) (Müller and Wickham, 2020) in R.

To identify microbiome features that characterized the differences within each lake, we performed linear discriminant analysis (LDA) using LEfSe (LDA Effect Size) (Segata et al., 2011) within the Galaxy web application and workflow framework of the Huttenhower laboratory.^2^ The LEfSe analysis comprises three steps. First, it uses the non-parametric factorial Kruskal-Wallis sum-rank test to identify discriminant features (ASVs) with significant differential abundances between the four Stuckberry Valley lakes. Second, to investigate the biological consistency of these features, pairwise tests are performed using the unpaired Wilcoxon rank-sum test. Finally, a LDA estimates the effect size of each differentially abundant feature. The Kruskal-Wallis, Wilcoxon, and LDA test cut-offs were set at 0.05, 0.05, and 2.0, respectively (the default values).

Community-wide diversity (β diversity) was calculated with the Jenson-Shannon divergence (JSD) method (phyloseq{}) using a filtered relative abundance ASV table (ASVs with a mean relative abundance across all samples < 0.00001% were rejected). Vectors showing limnological characteristics within the water columns were calculated with vegan{} (v.2.5.6) (Oksanen et al., 2019). Metadata collinearity was verified by the use of backward selection and the *vif.cca()* function. Adjusted R^2^ values were calculated with the *RsquareAdj()* function. They were visualized along with β diversity in Constrained Analysis of Principal Coordinates (CAP) using Jenson-Shannon divergence with ggplot2{}, as well as through a redundancy analysis with water chemistry parameters using Bray-Curtis dissimilarity. Groups were compared by permutational analysis of variance with *adonis()*, and within-group dispersion homogeneity was verified with *betadisper()*, both functions in the vegan package.

Statistical significance was determined using α = 0.05, and only statistically significant results are reported, unless stated otherwise.

## Data Availability Statement

The datasets presented in this study can be found in online repositories. The names of the repository/repositories and accession number(s) can be found below: https://www.ncbi.nlm.nih.gov/, PRJNA726255.

## Author Contributions

CM conceived the study, with input from DA, CG, and AC. CM, YK, and DA conducted the fieldwork and sampling. CM processed the samples and performed the lab work and the data analyses, with assistance from YK and CG. CM wrote the first draft of the manuscript which was edited and revised by CM, DA, CG, AC, and WV. All the authors approved the submitted version.

## Conflict of Interest

The authors declare that the research was conducted in the absence of any commercial or financial relationships that could be construed as a potential conflict of interest.

## Publisher’s Note

All claims expressed in this article are solely those of the authors and do not necessarily represent those of their affiliated organizations, or those of the publisher, the editors and the reviewers. Any product that may be evaluated in this article, or claim that may be made by its manufacturer, is not guaranteed or endorsed by the publisher.

## Footnotes

1
http://climate.weather.gc.ca

2
https://huttenhower.sph.harvard.edu/galaxy/
