# Supplementary material for: Local Habitat Filtering Shapes Microbial Community Structure in Four Closely Spaced Lakes in the High Arctic
Source: Front Microbiol. 2022 Feb 11;13:779505. doi: 10.3389/fmicb.2022.779505 (PMC8873593; doi:10.3389/fmicb.2022.779505)
Supplement: Supplementary file 1 [file Table_1.DOCX]

**SUPPLEMENTARY MATERIAL**

Supplementary material contains:

Fig. S1. Limnological profiles of the four Stuckberry Valley lakes

Table S1. Number of reads after treatment through the DADA2 pipeline

Fig. S2. Relative abundance of orders in the classes *Delta-* and *Gammaproteobacteria*

Fig. S3 RDA ordination

**TOP**

**BOTTOM**

**2FB**

**Y**

**Deep lakes**

**Shallow lakes**


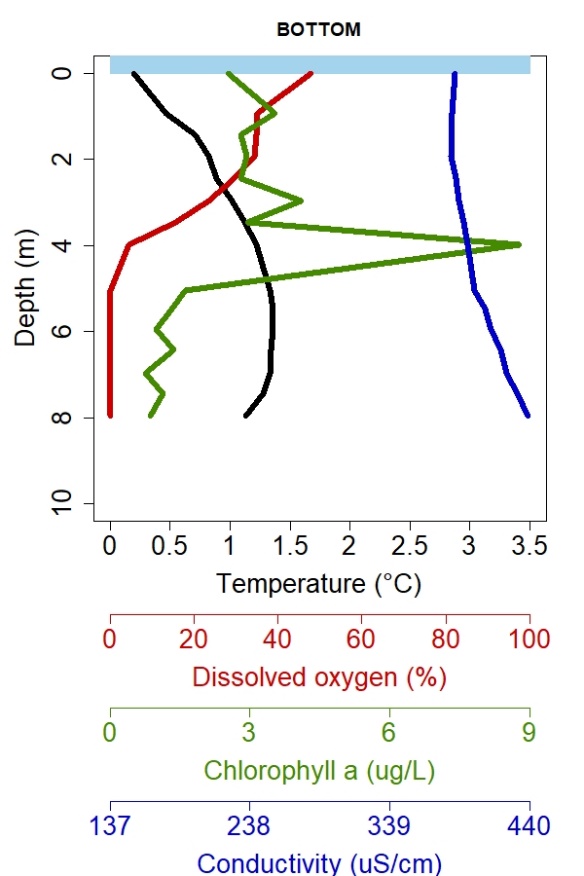

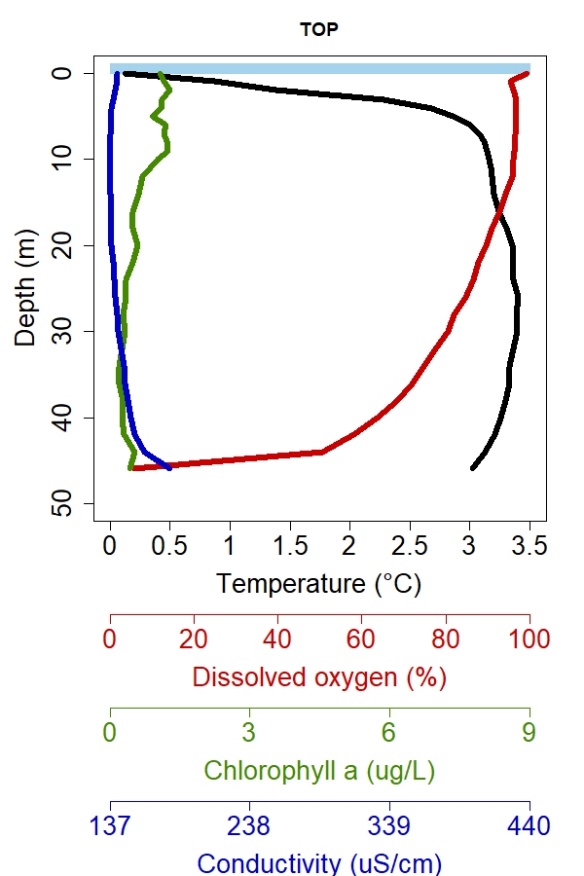

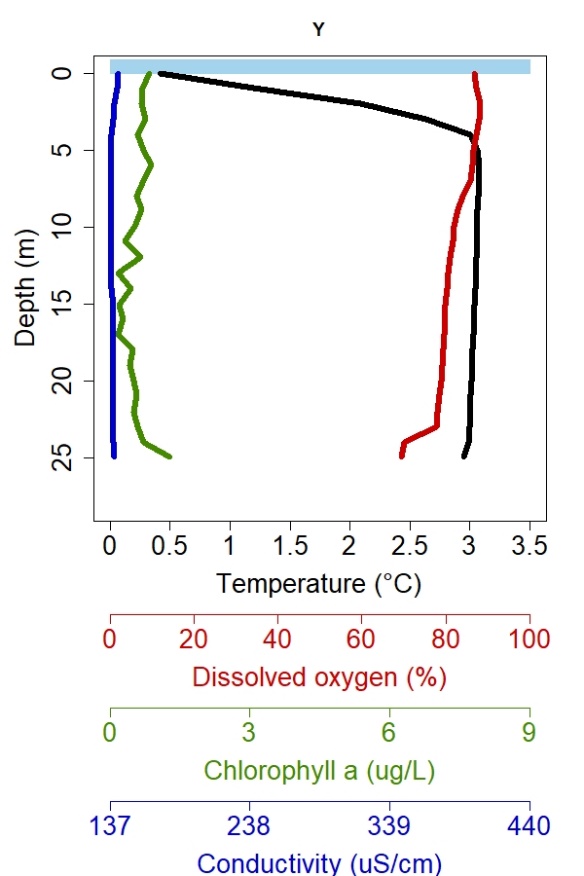

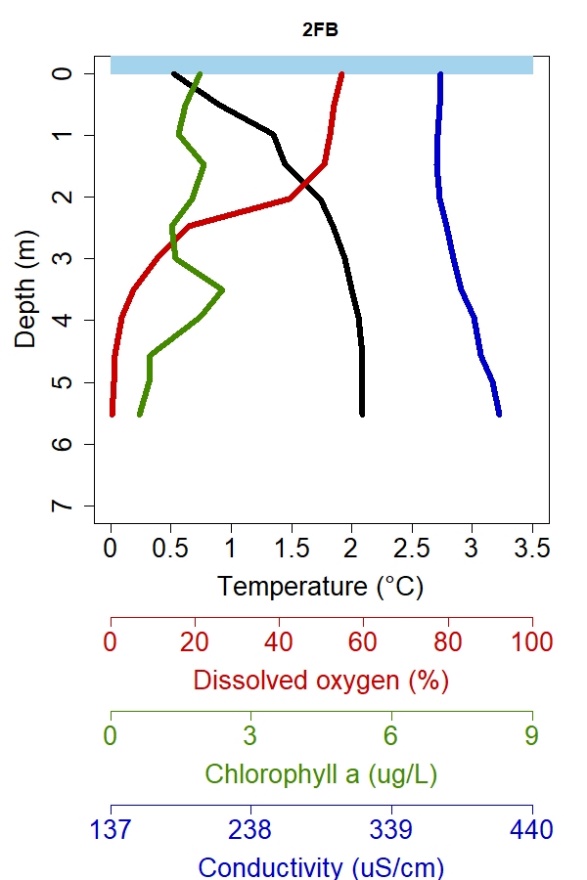


**Fig. S1. Limnological profile of the four Stuckberry Valley lakes.** The water column of the four Stuckberry Valley lakes had chemical and physical gradients. Lakes formed two groups based on limnological properties (deep vs. shallow).The light blue rectangle represents the ice thickness (1.11 m for Top and 0.90 m for Y, 2FB, and Bottom lakes). Note the changing graduations of the y-axis established according to the maximum depth of the four lakes.

**Table S1****. Number of reads after treatment through the DADA2 pipeline.** DNA concentrations (DNA, ngµL^-1^) were quantified using the Qubit 3.0 Fluorometer (Thermo Fisher Scientific). Lines in grey are removed samples due to sequencing issues. The total sequencing yield was 8,447,001 reads. A total of 4155 ASVs were identified among the samples.

**
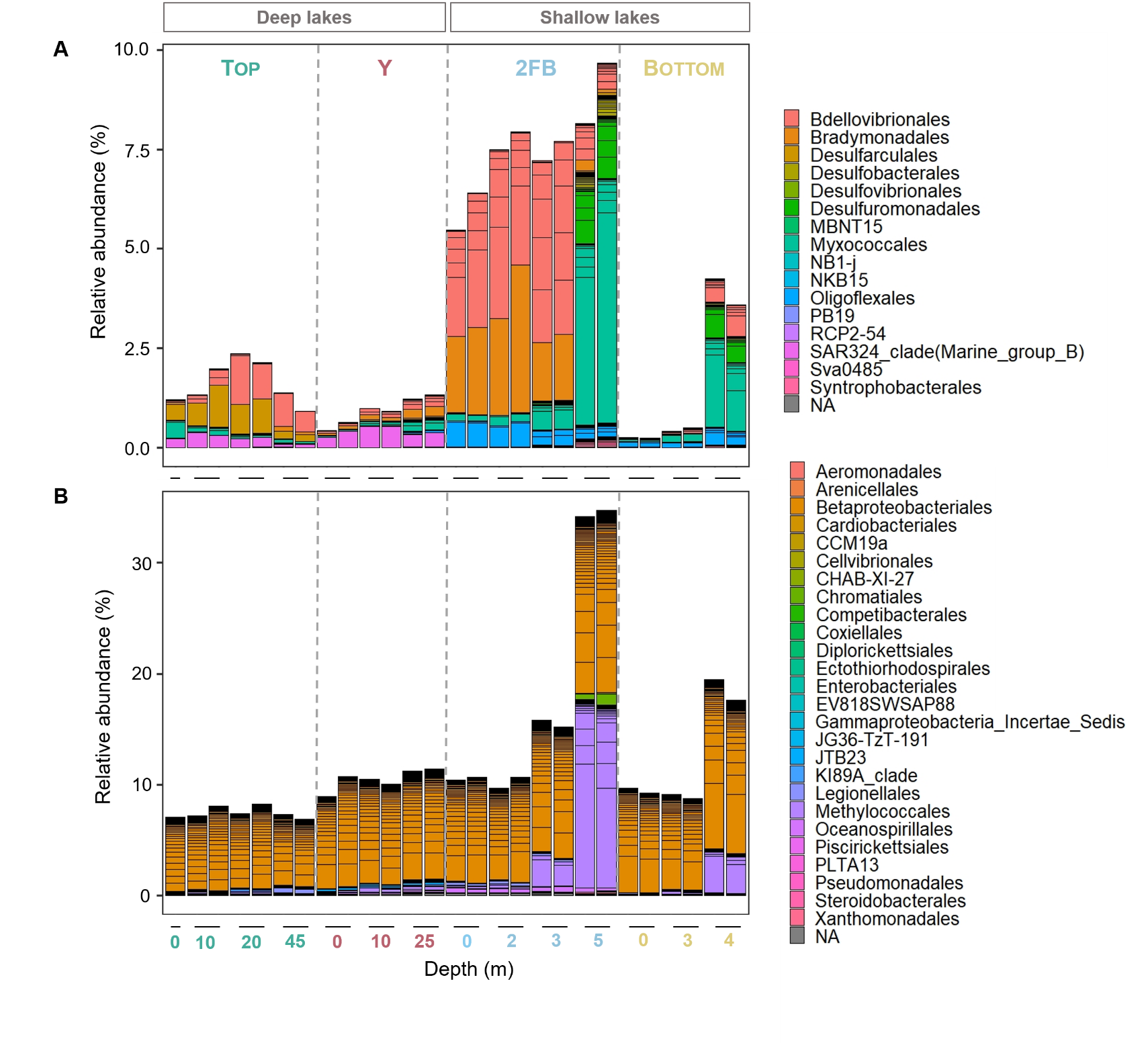
**

**B**

**Fig. S2. Relative abundance of orders in the classes *Delta-* and *Gammaproteobacteria*** (A) *Deltaproteobacteria* (B) *Gammaproteobacteria*

**Fig. S3. RDA ordination bi-plot revealing the relationships between environmental parameters (vectors) and microbial communities for each lake.**
